# LncRNA SNHG6 Upregulates KPNA5 to Overcome Gemcitabine Resistance in Pancreatic Cancer via Sponging miR-944

**DOI:** 10.3390/ph16020184

**Published:** 2023-01-25

**Authors:** Ge Gao, Xin Li, Hui Wu, Ling-li Huang, Yu-xin Lin, Zhi Huo, Zhong-yuan Xiang, Xiao Zhou

**Affiliations:** 1 Department of Clinical Laboratory Medicine, The Third Xiangya Hospital, Central South University, Changsha 410013, China; 2 School of Basic Medical Sciences, Central South University, Changsha 410013, China; 3 Department of Laboratory Medicine, The Second Xiangya Hospital, Central South University, Changsha 410011, China

**Keywords:** LncRNA SNHG6, Gemcitabine resistance, KPNA5, miR-944, pancreatic cancer

## Abstract

Gemcitabine (GEM) is the gold-standard therapeutic regimen for patients with pancreatic cancer (PC); however, patients may receive limited benefits due to the drug resistance of GEM. LncRNA SNHG6 is reported to play key roles in drug resistance, but its role and molecular mechanism in PC remain incompletely understood. We found that LncRNA SNHG6 is drastically downregulated in GEM-resistant PC and is positively correlated with the survival of PC patients. With the help of bioinformatic analysis and molecular approaches, we show that LncRNA SNHG6 can sponge miR-944, therefore causing the upregulation of the target gene KPNA5. In vitro experiments showed that LncRNA SNHG6 and KPNA5 suppress PC cell proliferation and colony formation. The Upregulation of LncRNA SNHG6 and KPNA5 increases the response of GEM-resistant PANC-1 cells to GEM. We also show that the expression of KPNA5 is higher in patients without GEM resistance than in those who developed GEM resistance. In summary, our findings indicate that the LncRNA SNHG6/miR944/KPNA5 axis plays a pivotal role in overcoming GEM resistance, and targeting this axis may contribute to an increasing of the benefits of PC patients from GEM treatment.

## 1. Introduction

Pancreatic cancer (PC), most commonly pancreatic ductal adenocarcinoma (PDAC), has the poorest prognosis among any common solid malignancy. It is believed to be the most aggressive disease, with a 5-year overall survival (OS) of approximately 10%. The global burden of PC has drastically increased in the past few decades, and it has become the third leading cause of cancer-related death [1]. Due to the late presentation of the disease and the anatomically inaccessible location of the pancreas, early diagnostic biomarkers and routine examinations for PC are largely insufficient. Attributed to the development of diagnosis and therapy, the 5-year OS has developed from lower than 5% in the 1990s to 10% in western countries in 2019. Chemotherapy is the primary treatment for patients who develop nonresectable PC and undergo resection in addition to radiation [2]. Gemcitabine (GEM), FOLFIRINOX, and albumin-bound paclitaxel are the most common chemotherapy regimens [3]. These chemotherapy or adjuvant chemotherapy regimens increase the long-term survival of patients with PC after surgery; however, chemoresistance may rapidly occur, and the patients may eventually suffer from tumor recurrence [4].

GEM-based chemotherapy is a key treatment for PC patients. GEM is a synthetic nucleoside analog that discontinues DNA synthesis. GEM enters cells via equilibrative and concentrative nucleoside transporters (hENTs and hCNTs), while hENT1 plays a key role in GEM uptake [5]. After entering the cell, GEM is phosphorylated to its main active metabolite 2′,2′-difluorodeoxycytidine triphosphate (dFdCTP) by deoxycytidine kinase; therefore, it competes with deoxycytidine triphosphate and takes action to inhibit DNA polymerase [6]. Despite being the cornerstone of PC therapy, the overall benefits in clinical practice are rather limited due to drug resistance. Numerous studies illustrate the potential mechanisms underlying GEM resistance, among which epigenetic alterations have attracted increasing attention. Recently, long noncoding RNAs, circular RNAs, and other epigenetic modifications have been found to play key roles in GEM resistance [7,8,9]. It is well established that many mechanisms are closely involved in GEM resistance, in particular, the miRNA-related pathway is considered a pivotal mechanism in regulating GEM-resistant related pathways such as KRAS, PI3K-AKT, NF-kB, P53, and Hedgehog [10,11]. As a key regulator of miRNA, long noncoding RNAs (LncRNAs) are shown to be promising therapeutic targets. The administration of short interfering RNAs and antisense RNAs to antagonize the levels and activities of oncogenic LncRNAs or RNAs that increase the levels and activities of the tumor-suppressive LncRNAs have been shown to be promising to function as anticancer drugs [12]. Although recent years have seen several pioneering types of research, there are currently no established markers to predict the activity of GEM on PC.

LncRNAs are one of the most abundant RNAs that have no or limited protein-coding potential [13]. LncRNAs are usually more than 200 nucleotides in length and are reported to be aberrantly expressed in PC [14]. Although the function of LncRNAs is not fully understood, they are reported to play critical roles in numerous biological processes, such as epigenetic regulation, RNA decay, alternative splicing, and chromosomal accessibility [15]. More recently, LncRNAs have been found to play a key role in cell cycle control, cell differentiation, cancer metastasis, and drug resistance in PC, highlighting the importance of LncRNAs in the development and treatment of PC [16]. In addition, LncRNAs are emerging as biomarkers for the diagnosis and treatment of PC [15]; however, the complete role and regulatory mechanism of LncRNAs in GEM resistance remain largely unknown.

LncRNA SNHG6 (Small Nucleolar RNA Host Gene 6) is aberrantly expressed in several types of cancer, such as hepatocellular carcinoma and colorectal cancer [17,18]. The host gene SNHG6 has long been considered a putative oncogene owing to its extensive involvement in many processes of tumorigenesis and metastasis [19]. LncRNA SNHG6 is dysregulated in cancer, and its copy number displays huge variations compared to normal tissue, making LncRNA SNHG6 a promising biomarker in cancer diagnosis [17,20]. LncRNA SNHG6 can function as a miRNA sponge, thus linking the mRNA network and transcription factors downstream to play pivotal roles in regulating numerous cellular functions [21]. LncRNA SNHG6 is reported to be highly expressed and act as an oncogenic LncRNA in the carcinogenesis of lung adenocarcinoma, glioma, breast cancer, colorectal cancer, and hepatocellular carcinoma by sponging the miRNAs [22]; it also functions as a tumor-suppressive molecule to inhibit cell proliferation and metastasis in colorectal cancer and gastric cancer [23,24]. Although the role of LncRNA SNHG6 in several types of cancer has been extensively studied, its expression and function in PC remain largely uncertain.

MiR-944 is abnormally expressed in cancers in multiple systems, including neural, endocrine, respiratory, reproductive, and digestive systems. miR-944 is also associated with the development of Paclitaxel (PTX), Cisplatin (DDP), Rapamycin (RAPA), and Doxorubicin (DOX) resistance in cancer cells [25]. PTX is a member of the paclitaxel family, and DDP interacts with purine bases on DNA to induce cell apoptosis, which is similar to GEM [26], suggesting that targeting miR-944 may overcome GEM resistance.

Karyopherin alpha (KPNA) 5 is known as a protein cargo to import proteins into the nucleus [27]. KPNA5 functions as an adaptor molecule that carries protein cargoes carrying the nuclear localization signal (NLS) and Karyopherin beta (KPNB) from the cytoplasm to the nucleus [28]. Furthermore, KPNA5 could also mediate nuclear membrane formation, protein degradation, gene expression, cytoplasmic retention, and mRNA-related function to participate in numerous bioprocesses [29]. In many cancers, KPNA5 is highly expressed and interacts with the tumor-suppressor gene PHB2 [30,31]. On the other side, KPNA5 is reported to be correlated with a poor prognosis in patients with lung adenocarcinoma, and it is tightly involved in the regulation of cell cycle checkpoints and the cell mitotic cycle [28]. Along with Cdc14B, ESCO1, MAP3K3, and CSIG, KPNA5 is found to be highly expressed and regulates cell cycle and senescence progression [32]. Due to limited research on KPNA5 in cancer, the expression and function of KPNA5 in PC have yet to be discovered; in particular, whether KPNA5 is related to GEM resistance is of particular interest to us.

In this study, we found that a high expression of LncRNA SNHG6 is associated with better prognosis and is downregulated in GEM-resistant PC. We also found that LncRNA SNHG6 can act as a microRNA sponge to silence miR-944, which causes an increased expression of KPNA5. We show that the LncRNA SNHG6/miR-944/KPNA5 axis plays a key role in overcoming GEM resistance in PC, which provides new insights into enhancing the GEM response of PC by targeting the LncRNA SNHG6/miR-944/ KPNA5 axis.

## 2. Results and Discussion

### 2.1. LncRNA SNHG6 Is Downregulated in GEM-Resistant PC

**Figure 1:**
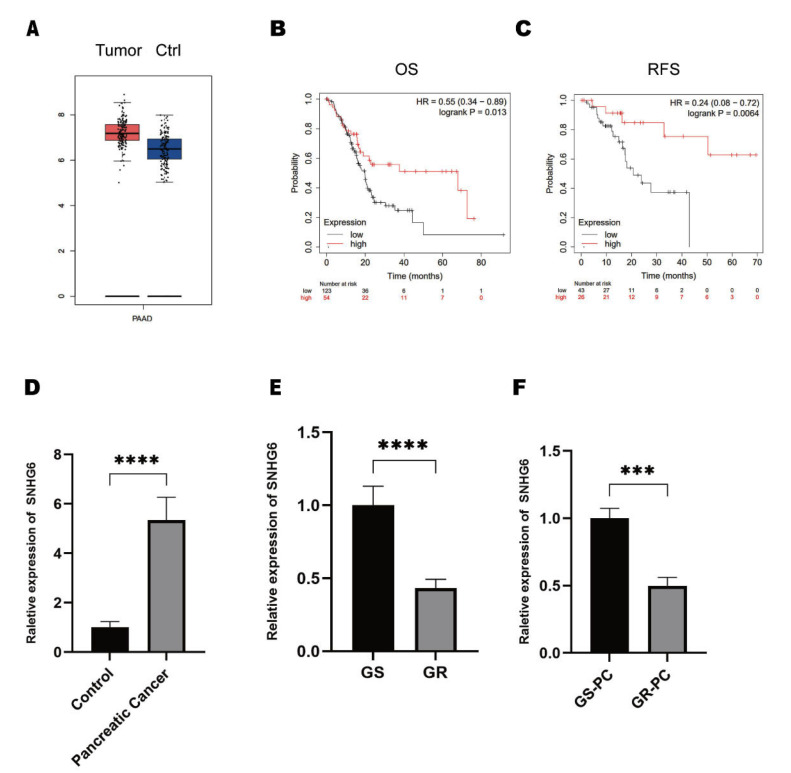
LncRNA SNHG6 **is dysregulated in PC.** (**A**) Expression of LncRNA SNHG6 in PC, analyzed by the online toolkit GEPIA while LncRNA SNHG6 and pancreatic cancer were made inquiries. (**B**) The correlation of LncRNA SNHG6 and the overall survival rate of PC patients were analyzed by the online database Kaplan–Meier plotter; OS, overall survival; RFS, recurrence-free survival; HR, hazard ratio. (**C**) The correlation of LncRNA SNHG6 and the recurrence-free survival rate of PC patients. (**D**) The expression of LncRNA SNHG6 in the resected PC tissues and para-carcinoma tissues (control), quantified by RT-PCR, *n* = 3, ****, *p* < 0.0001. (**E**) RNA level of LncRNA SNHG6 in GEM-resistant (GR) and the GEM-sensitive (GS) PANC-1 cells, quantified by RT-PCR, *n* = 3, ****, *p* < 0.0001. (**F**) Expression level of LncRNA SNHG6 in the resected PC tissue from GEM-resistant PC patients (GR-PC) and GEM-sensitive PC patients (GS-PC), *n* = 3, ***, *p* < 0.001.

The dysregulated expression of LncRNA SNHG6 has been broadly reported in several types of cancer, such as colorectal cancer, hepatocellular carcinoma, and chondrosarcoma [18,19,33]. However, the expression of LncRNA SNHG6 in PC remains unknown. We applied the online toolkit GEPIA to check the expression of LncRNA SNHG6 in PC [34]. We found that the LncRNA SNHG6 was upregulated in patients with PC compared with controls (Figure 1A). Then, we examined the overall survival (OS) and recurrence-free survival (RFS) rates of patients with PC. Unexpectedly, we found that patients with high expressions of LncRNA SNHG6 had better OS and RFS rates (Figure 1B,C). Considering that GEM is the first-line therapeutic regimen and commonly leads to drug resistance, we wondered whether these findings were due to the role of LncRNA SNHG6 in GEM resistance. Therefore, we first checked the expression of LncRNA SNHG6 in PC tissue. We first extracted total RNA from the pancreatic cancer tissue and para-carcinoma tissue, and we detected the expression of LncRNA SNHG6 in each group by using RT-PCR. We found that LncRNA SNHG6 is indeed upregulated in PC, which is consistent with GEPIA (Figure 1D). Afterward, we compared the expressions of LncRNA SNHG6 between the PC cell line PANC-1 and its GEM-resistant form PANC-1. We extracted total RNA from GEM-sensitive and GEM-resistant PANC-1 cells and performed RT-PCR, and we found that the LncRNA SNHG6 expression was significantly lower in PANC-1-GEM cells than in PANC-1 cells (Figure 1E). Furthermore, we checked the expression of LncRNA SNHG6 in the tissues of PC patients. We collected clinical resected tumor tissues from patients who were GEM-sensitive and those who had developed GEM resistance but had not received other chemotherapy yet. Total RNA was extracted and RT-PCR was used to check the expression of LncRNA SNHG6. We found that LncRNA SNHG6 was significantly downregulated in GEM-resistant patients compared with those who had not yet developed GEM resistance (Figure 1F). These findings lead us to propose a putative role for LncRNA SNHG6 in regulating GEM resistance in PC.

### 2.2. Upregulation of LncRNA SNHG6 Increases the Response of PC Cells to GEM

**Figure 2:**
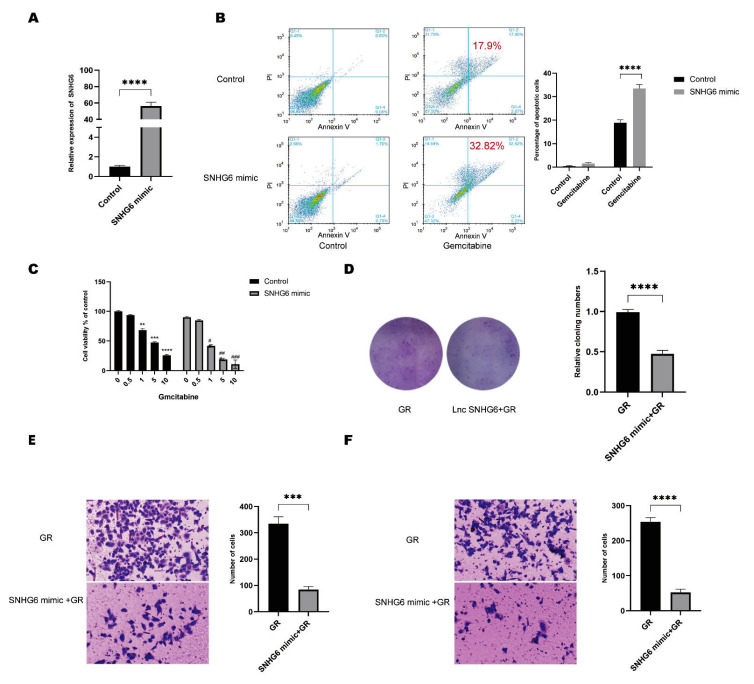
**LncRNA SNHG6 participates in GEM resistance of PC.** (**A**) RT-PCR quantification of the expression level of LncRNA SNHG6 after PANC-1-GEM cells were transfected with LncRNA SNHG6 mimics, *n* = 3, ****, *p* < 0.0001. (**B**) FACS analysis of apoptosis of PANC-1 cells after LncRNA SNHG6 transfection, and histogram of quantification of the apoptotic cell percentage. *n* = 3, ****, *p* < 0.0001. (**C**) CCK8 detection of the viability of LncRNA SNHG6-overexpressing PANC-1 cells treated with GEM; *, compared with the group “0”, *, *p* < 0.05, **, *p* < 0.01, ***, *p* < 0.001, ****, *p* < 0.0001; #, compared with control, #, *p* < 0.05, ##, *p* < 0.01, ###, *p* < 0.001. (**D**) Colonies formed by GEM-resistant PANC-1 cells transfected with LncRNA SMHG6 mimic or control, and histogram of quantification of cloning numbers. *n* = 3, ****, *p* < 0.0001. (**E**,**F**) Images of cells invaded through the matrix-coated transwell and migrated after transfection with LncRNA SMHG6 mimic or control, and histogram of quantification of the number of cells, *n* = 3, ***, *p* < 0.001, ****, *p* < 0.0001. The cells in the inner chamber were wiped off and the outer layer of cells was fixed with 4% paraformaldehyde and then stained with crystal violet.

To ensure the function of LncRNA SNHG6 in GEM resistance, we overexpressed LncRNA SNHG6 by transfecting cells with an expression vector encoding LncRNA SNHG6 mimics in PANC-1-GEM cells (Figure 2A). We first stained the cells with Annexin V-FITC and PI and detected the apoptosis of cells after GEM treatment via flow cytometry (FACS); we found that in the LncRNA SNHG6 overexpressed cells, 1 μg/mL GEM can induce 32.82% cell apoptosis, which is significantly higher than that in the control (Figure 2B). Then, cell viability tests were carried out using a CCK8-based cell viability assay to check the influence of GEM on PC cell viability. We found that cells overexpressing LncRNA SNHG6 showed much lower viabilities compared with the control at the same concentrations of GEM (Figure 2C). Additionally, PANC-1-GEM cells displayed a reduced capability to form colonies when LncRNA SNHG6 was highly expressed, suggesting that PC cells with an overexpression of LncRNA SNHG6 have lower proliferation capacities and are more sensitive to GEM treatment (Figure 2D). Considering the aggressive potential of PC, we also checked the role of LncRNA SNHG6 in regulating invasion. We found that cells with LncRNA SNHG6 overexpression showed lower invasion capabilities (Figure 2E,F). These findings insure us that LncRNA SNHG6 plays an unneglectable role in overcoming GEM resistance; however, the mechanisms remain to be further studied.

### 2.3. LncRNA SNHG6 Upregulates KPNA5 Expression by Acting as a Sponge of miR-944

**Figure 3:**
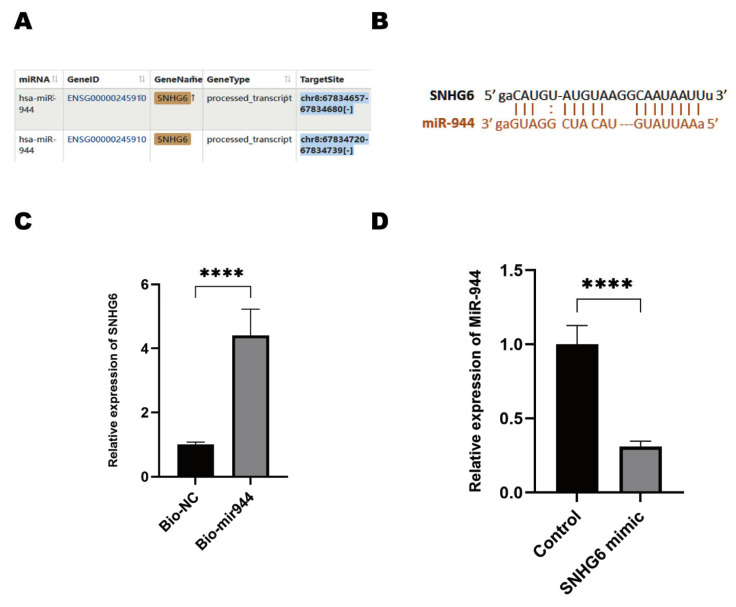
LncRNA SNHG6 **acts as a sponge to interact with miR-944.** (**A**) Scheme of the predicted interaction of LncRNA SNHG6 with miR-944. (**B**) ENCORI prediction of the binding sites between LncRNA SNHG6 and miR-944. (**C**) Histogram of the PCR assay detecting LncRNA SNHG6 pulled down by biotinylated miR-944 (bio-miR-944) or the control (bio-NC), *n* = 3, ****, *p* < 0.0001. (**D**) PCR measurement of miR-944 in PANC-1 cells that were transfected with LncRNA SNHG6 mimics expressing vectors or control, *n* = 3, ****, *p* < 0.0001.

It is well accepted that LncRNAs commonly act as competing endogenous RNAs (ceRNAs) to diminish the silencing functions of microRNAs [18], therefore, we speculated that LncRNA SNHG6 serves as a microRNA sponge to overcome GEM resistance. To determine whether LncRNA SNHG6 acts as a ceRNA, we used the online databases ENCORI (https://starbase.sysu.edu.cn/, accessed on 3 September 2022). and LncRNASNP2.0 (http://bioinfo.life.hust.edu.cn/LncRNASNP#!/, accessed on 3 September 2022.) to discover potent targeted miRNAs. We used the miRNA-LncRNA database and input miR-944 as an inquiry. We found that LncRNA SNHG6 interacts with miR-944 (Figure 3A). MiR-944 has been largely shown to play broad functions in cancer. In addition to its function as a tumor-suppressing gene [35], miR-944 has also been shown to be associated with a poor prognosis and shorter OS [36]. However, the expression of miR-944 and its role in PC tumorigenesis and drug resistance are unclear. We checked the binding sites between LncRNA SNHG6 and miR-944 by the online toolkit ENCORI, and we found proper interaction between LncRNA SNHG6 and miR-944 (Figure 3B). Due to the fact that it has been reported that LncRNA SNHG6 interacts with AGO2 [17,37], we directly used biotinylated miR-944 to pull down RNAs in PANC-1 cells, and we confirmed that LncRNA SNHG6 can largely bind with miR-944 as we found LncRNA SNHG6 in the miR-944 pull-down portion (Figure 3C). To further verify whether LncRNA SNHG6 acts as a miRNA sponge, we measured miR-944 in PANC-1 cells overexpressing LncRNA SNHG6 and found that miR-944 was drastically decreased by LncRNA SNHG6 overexpression (Figure 3D). These results suggest that LncRNA SNHG6 may function as an endogenous competing RNA by sponging miR-944.

### 2.4. LncRNA SNHG6/miR-944 Regulates the Expression of KPNA5

**Figure 4:**
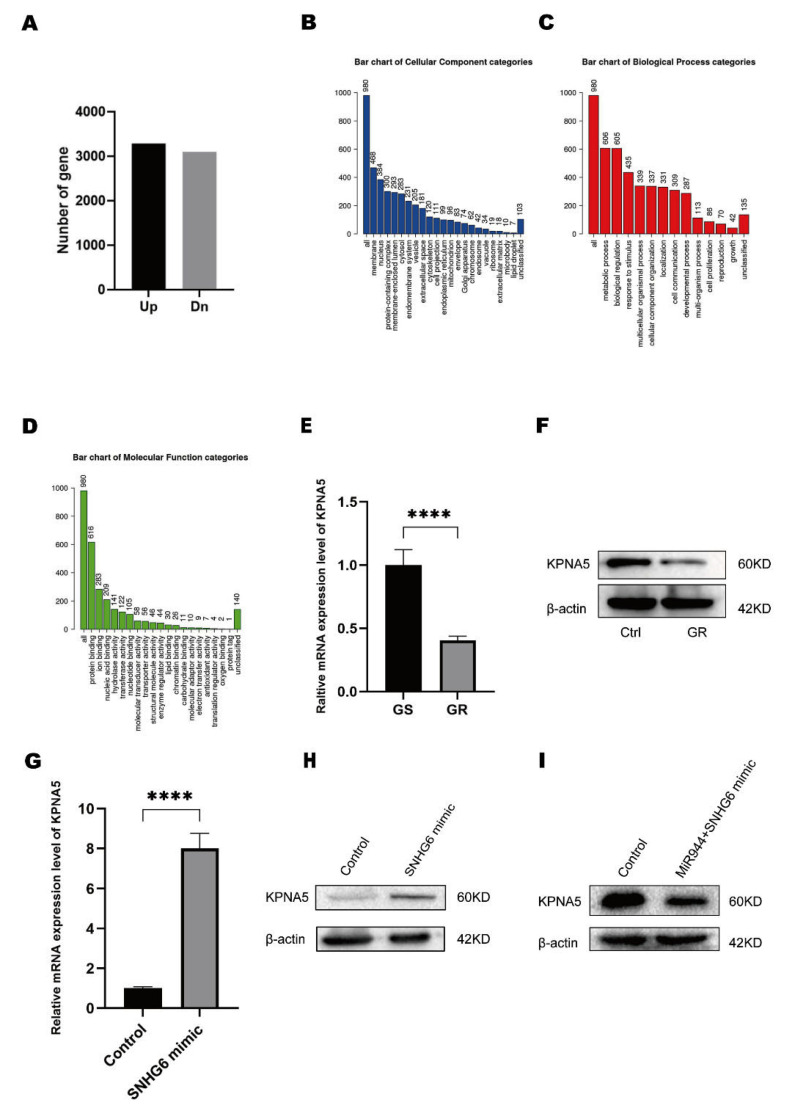
LncRNA SNHG6 **regulates the expression of KPNA5 in PC.** (**A**) Histogram of gene expression detected by RNA CHIP measurement after LncRNA SNHG6 overexpression in PANC-1 cells. (**B**) Enrichment of categories of cellular components in which the upregulated genes were enriched and analyzed by the online toolkit WebGestalt. (**C**) Histogram of categories of a biological process in which the upregulated genes were enriched by WebGestalt. (**D**) Histogram of categories of molecular function in which the upregulated genes were enriched by WebGestalt. (**E**) PCR analysis of KPNA5 levels in GEM-sensitive (GS) and GEM-resistant (GR) PANC-1 cells, *n* = 3, ****, *p* < 0.0001. (**F**) Western blotting analysis of KPNA5 expression in GEM-sensitive (Ctrl) and GEM-resistant (GR) PANC-1 cells. (**G**) PCR analysis of KPNA5 levels in PCNC-1 cells transfected with LncRNA SNHG6 mimic or control, *n* = 3, ****, *p* < 0.0001. (**H**) Western blotting analysis of KPNA5 expression in PCNC-1 cells transfected with LncRNA SNHG6 mimic or control. (**I**) Western blotting analysis of KPNA5 expression in PCNC-1 cells co-transfected with LncRNA SNHG6 mimic and miR-944 or control.

We sought to confirm the downstream targets of LncRNA SNHG6 and miR-944. We first mapped the differentially expressed mRNAs in LncRNA SNHG6-overexpressing cells by RNA-Chip assay. We found that 3285 mRNAs were upregulated and 3097 mRNAs were downregulated (Figure 4A). We performed a gene ontology (GO) analysis by using the WebGestalt tool [38] and found that the top three enriched cellular components (CCs) were the membrane, nucleus, and membrane-enclosed lumen (Figure 4B). In addition, the three best-enriched biological processes (BP) were the metabolic process, biological regulation, and response to stimulus (Figure 4C), and the most enriched molecular functions (MF) were protein binding, ion binding, and nucleic-acid binding (Figure 4D). Metabolism was the most enriched MF, among which KPNA5 showed an RPKM larger than 1638; thus, we speculate that KPNA5 is upregulated by LncRNA SNHG6 in PC. We performed RT-PCR to check the expression of LncRNA SNHG6 and found that KPNA5 was highly downregulated in PANC-1-GEM cells (Figure 4E,F). After LncRNA SNHG6 overexpression, KPNA5 was upregulated at both the mRNA and protein levels (Figure 4G,H). To test whether KPNA5 is a confident target of miR-944, we predicted the targets of miR-944 with the online tools. Both TargetScan and ENCORI showed that miR-944 binds with the 3′UTR of KPNA5 properly (Supplementary Figure S1A,B). It was proved by other groups that KPNA5 displays several binding sites with AGO1/2, which is the key player in the RNA-induced silencing complex [39,40]. In addition, we overexpressed miR-944 in LncRNA SNHG6-overexpressing PANC-1 cells, and the expression level of KPNA5 was reduced (Figure 4I), which is consisted with the analysis or ENCORI that miR-944 is negatively correlated with the KPNA5 level in pan-cancer (Supplementary Figure S1C). These findings show that KPNA5 is a key protein of the LncRNA SNHG6/miR-944 axis and that LncRNA SNHG6/miR-944/KPNA5 plays a pivotal role in overcoming GEM resistance.

### 2.5. KPNA5 Helps Overcome GEM Resistance in Patients with PC

**Figure 5:**
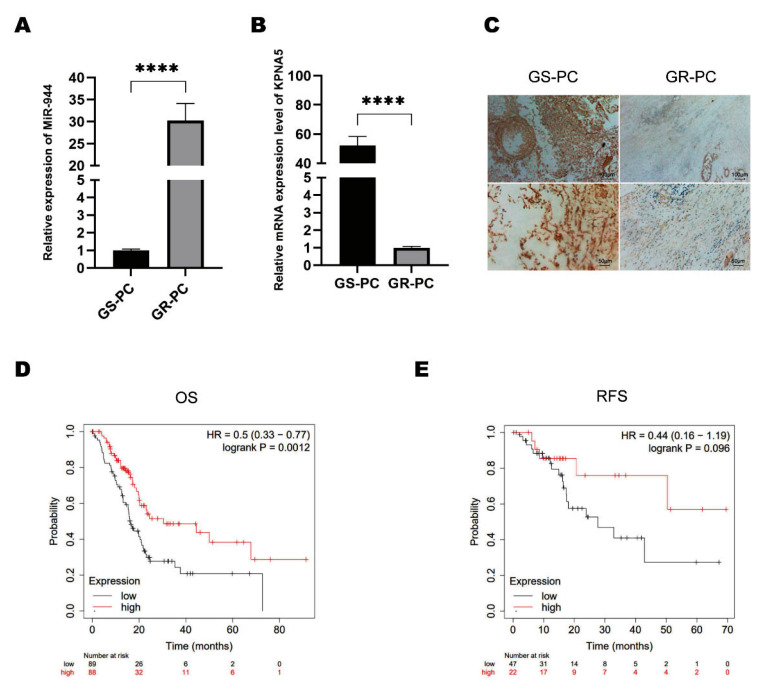
**miR944-mediated KPNA5 expression participates in GEM resistance in PC.** (**A**) PCR measurement of the expression of miR-944 in GEM-sensitive (GS-PC) and GEM-resistant (GR-PC) PC patients, *n* = 3, ****, *p* < 0.0001. (**B**) PCR measurement of KPNA5 mRNA levels in GEM-sensitive (GS-PC) and GEM-resistant (GR-PC) PC patients, *n* = 3, ****, *p* < 0.0001. (**C**) Immunohistochemistry staining of KPNA5 in tumor tissue from GEM-sensitive and GEM-resistant PC patients. (**D**) Correlation of KPNA5 levels with the OS in PC patients. (**E**) Correlation of KPNA5 level with recurrence-free survival rate in PC patients.

After we confirmed the expression of the LncRNA SNHG6/miR-944/KPNA5 axis, we wondered whether this mechanism is the reason why patients with high LncRNA SNHG6 expression have a better prognosis. To confirm this hypothesis, we first checked the expressions of miR-944 in GEM-sensitive and GEM-resistant PC patients by RTPCR. We found that miR-944 was significantly lower in GEM-sensitive patients than in GEM-resistant patients (Figure 5A). Consistent with this finding, we performed immunohistochemistry staining to check the protein levels in the tissues of PC patients. We confirmed that the KPNA5 expression was higher at the protein level in GEM-sensitive patients (Figure 5B,C). After we checked the expression of KPNA5 in patients with PC, we analyzed the correlation of KPNA5 with the OS and RFS of patients with PC by Kaplan–Meier plotter. We found that high KPNA5 expression was associated with a higher OS and RFS of PC patients (Figure 5D,E), indicating that LncRNA SNHG6-mediated KPNA5 plays a key role in overcoming GEM resistance and may predict a better prognosis of PC.

## 3. Materials and Methods

### 3.1. Biological Samples

Tumor tissues from PC patients were obtained from surgical resection at the Third Xiangya Hospital between July 2021 and February 2022. Tissue samples were assessed by one experienced pathologist blinded to the clinical information, and the pathologic diagnosis was made. Total RNA extraction from the specimens was performed using a RNA extraction kit (AM1912, Thermo Fisher, Waltham, MA, USA). Protein was extracted by RIPA buffer supplemented with proteinase inhibitor (5871, Cell Signaling Technology, Danvers, MA, USA). This study was approved by the Human Ethics Review Committee of the Third Xiangya Hospital (Receipt number: 2022-S044).

### 3.2. Cell Culture

The human PDAC cell lines PANC-1 and GEM-resistant PANC-1 (PANC-1-GEM) were purchased from the American Type Culture Collection. Cells were cultured in DMEM (12430047, Gibco, Waltham, MA, USA) supplemented with 10% fetal bovine serum (10099141C, Gibco, Waltham, MA, USA), 1% penicillin, and 1% streptomycin. Cells were maintained in an incubator with 5% CO2 at 37  °C.

### 3.3. Transfection

The transient expression of lncSNHG6 and miR-944 was achieved using LncRNA SNHG6 or miR-944 mimic oligonucleotide. Control-RNA oligonucleotides were designed and synthesized. Lipofectamine 3000 RNA Transfection Reagent (L3000001, Thermo Fisher Scientific, Waltham, MA, USA) was used according to the manufacturer’s instructions. Cells were harvested for further experiments after 48 h of cultivation.

### 3.4. Bioinformatic Analysis

A WEB-based GEne Set Analysis Toolkit (WebGestalt, http://www.webgestalt.org/, accessed on 20 August 2022) was used to analyze the Gene Ontology enrichment, including cellular components, biological process, and molecular-function categories. In addition, Kaplan–Meier Plotter (http://kmplot.com/analysis/, accessed on 20 August 2022) was employed to analyze PC overall survival and recurrence-free survival rates as described previously [41]. TargetScan (https://www.targetscan.org/vert_72/, accessed on 12 January 2023) and ENCORI (https://starbase.sysu.edu.cn/index.php, accessed on 12 January 2023) were used to predict the binding sites of RNAs. The Kaplan–Meier survival analysis with the log-rank test was also used to compare the survival difference between the above two groups. For Kaplan–Meier curves, *p*-values and a hazard ratio (HR) with 95% confidence interval (CI) were generated by log-rank tests and univariate Cox proportional hazards regression.

### 3.5. Quantitative Polymerase Chain Reaction (qPCR)

qPCR was performed as previously described [42]. The primer pairs are shown in Table 1. In brief, RNA was reverse-transcribed using a HiScript II 1st Strand cDNA Synthesis Kit (R211-02, Vazyme, Nanjing, China). For qPCR of miRNA, cDNA was synthesized using an All-in-One™ miRNA First-Strand cDNA Synthesis Kit (QP017-GC, GeneCopoeia, Rockville, MD, USA). qPCR was performed using SYBR qPCR Master Mix (Q711, Vazyme, Nanjing, China) on an ABI Stepone Plus system. The LncRNA and mRNA levels were normalized to 18S rRNA. The miRNA level was normalized against U6 small nuclear RNA, all primers were synthesized by Sango BioTech(Shanghai, China).

### 3.6. Western Blotting

Western blotting was performed as described in a previous study [43]. In short, proteins were separated in a 10% SDS–PAGE gel and transferred to a 0.22-μm PVDF membrane. Afterward, the membrane was incubated with primary antibodies at 4 °C overnight. After 3 washes, secondary antibodies were added to the membrane and incubated for 1 h at RT. An ECL system (32106, Thermo Scientific, Waltham, MA, USA) was used to initiate and observe a luminous reaction. ImageJ software was used to calculate the grayscale values.

### 3.7. RNA Pulldown

RNA pull-down was performed as Ye et al. described [44]. PANC-1 cells were transfected with biotinylated miRNA (50 nM). After 48 h of cultivation, the cells were harvested and lysed with TRIzol. The cell lysates were incubated with M-280 streptavidin magnetic beads (11205D, Invitrogen, Waltham, MA, USA) following the manufacturer’s instructions. The miRNA-binding RNAs were purified and processed for further qRT—PCR analysis.

### 3.8. Colony Formation

PANC-1 cells were transfected with the desired LncRNA SNHG6 and miR-944 and their corresponding controls. Afterward, the cells were evenly spread into 6-well plates. The cells reached a density of 500 cells per well for each well. After 7 days of cultivation, the cells were fixed with 4% formaldehyde and washed twice with PBS. Afterward, the cells were stained with 1% crystal violet (25% methanol) for 10 min at RT, photographed using a light microscope, and counted.

### 3.9. CCK8-Based Cell Viability Assay

PANC-1 cells were cultured overnight in 96-well plates at a density of 5×103 cells per well. The LncRNA SNHG6 mimic and its corresponding negative control were transfected. After 48 h, 10 μL of CCK-8 (BS350B, BIOSHARP, Tallinn, Estonia) reagent was added to each well, and the plates were further cultured for 4 h. Afterwards, the absorbance at 450 nm was measured on a TECAN microplate reader, and the viability was calculated accordingly.

### 3.10. Flow Cytometry Analysis

PANC-1 cells were transfected with the desired RNA and cultured overnight in 6-well plates at a density of 5 × 105 cells per well. After cultivation for 48 h, both adherent and floating cells were harvested and washed with PBS. After washing, the cells were stained with Annexin V-FITC and PI (C1062S, Beyotime, Nantong, China) for 10 min at RT. The subsequent analysis was performed on a flow cytometer (Mindray) and analyzed with the configured program.

### 3.11. Transwell-Based Cell Invasion Assay

After transfection with the desired RNA or the corresponding negative control, PANC-1 cells were cultured for an additional 48 h and used for Transwell analysis. Transwell inserts were precoated with a Matrigel matrix (356234, Corning, Corning, New York, USA). The cells were inoculated into the upper chamber of transwell inserts at a density of 4 × 103 cells per well. The upper chamber was supplemented with 200 μL of serum-free medium, and the lower chamber was supplemented with 500 μL of medium supplemented with 10% FBS. After 72 h of culture, the cellular invasion was evaluated by staining with 0.5% crystal violet, and images were captured with microscopy.

### 3.12. Immunohistochemistry Staining

Anti-KPNA5 antibodies (1:250, PA5-84181, Invitrogen, Waltham, MA, USA) were used as the primary antibodies. IHC staining was performed as previously described [45]. Briefly, 5-μm-thick deparaffinized sections of FFPE were stained with the primary antibody. Antigens were retrieved by boiling tissue sections in 0.01 M citrate (pH 6.0). Endogenous peroxidase was blocked with 3% hydrogen peroxide for 15 min at RT. Slides were incubated with HRP-conjugated anti-rabbit secondary antibody at RT for 1 h. Diaminobenzidine was used as the chromogen.

## 4. Conclusions

**Figure 6:**
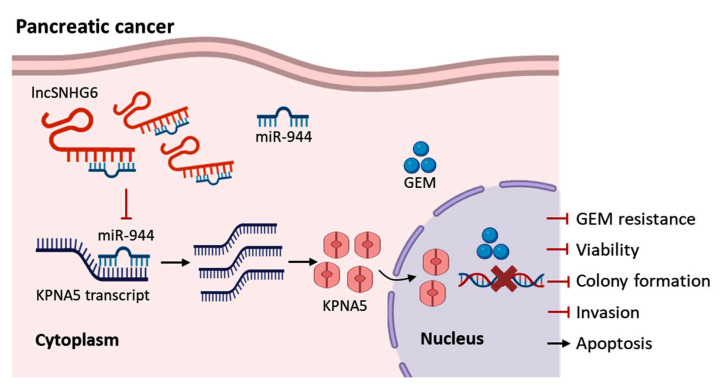
**Schematic illustration of the role of** LncRNA SNHG6**/miR-944/KPNA5 in overcoming GEM resistance in PC.** LncRNA SNHG6 is highly expressed to sponge and mediates silencing of miR-944, which leads to the elevated expression of KPNA5 protein. Activation of LncRNA SNHG6/miR-944/KPNA5 axis increases the sensitivity of PC to GEM, thus inducing cell apoptosis and reducing cell viability and colony formation and invasion during GEM treatment. Loss of LncRNA SNHG6 causes miR-944 elevation and KPNA5 reduction, which will result in GEM resistance of PC.

The LncRNA SNHG6 and KPNA5 are drastically decreased in GEM-resistant PC and are positively correlated with a better prognosis for PC patients. LncRNA SNHG6 sponges miR-944 to upregulate the expression of KPNA5, thereby functioning to suppress the proliferation and colony formation of PC cells. We show that LncRNA SNHG6 increases the response of GEM-resistant PANC-1 cells to GEM. Increasing the expression of LncRNA SNHG6 and KPNA5 can significantly increase the response of PANC-1-GEM to GEM. Our findings indicate that LncRNA SNHG6 may interact with miR-944 to regulate the expression of KPNA5, thus participating in overcoming GEM resistance in PC (Figure 6).

## Figures and Tables

**Table 1:** List of primers for real-time PCR.

GENE	Primer Sequence
SNHG6	Forward 5’-3’ TGCCAGCAGTGACAGCAGCA
	Reverse 5’-3’ TACGGAGGTGGAGTGCCAT
miR-944	Forward 5’-3’ GCACTCCTAAAATTATTGTACATCG
	Reverse 5’-3’ TATGGTTGTTCACGACTCCTTCAC
KPNA5	Forward 5’-3’ TCAGGAACAGGCTGTTTGGG
	Reverse 5’-3’ TGGGGTCATCTTCTTCTACAC
18S rRNA	Forward 5’-3’ TGTGCCGCTAGAGGTGAAATT
	Reverse 5’-3’ TGGCAAATGCTTTCGCTTT
U6	Forward 5’-3’ CTCGCTTCGGCAGCACATATACT Reverse 5’-3’ACGCTTCACGAATTTGCGTGTC

## Data Availability

Not applicable.

## Supplementary Materials

The following supporting information can be downloaded at: https://www.mdpi.com/article/10.3390/ph16020184/s1, Figure S1: The interactive relationship between miR-944 and KPNA5.
