# Supplementary material for: LncRNA SNHG6 Upregulates KPNA5 to Overcome Gemcitabine Resistance in Pancreatic Cancer via Sponging miR-944
Source: Pharmaceuticals (Basel). 2023 Jan 25;16(2):184. doi: 10.3390/ph16020184 (PMC9961296; doi:10.3390/ph16020184)
Supplement: Supplementary file 1 [file pharmaceuticals-16-00184-s001.zip › pharmaceuticals-2090482-supplementary.pdf]

pharmaceuticals

Article

LncRNA SNHG6 Upregulates KPNA5 to Overcome Gemcitabine Resistance in Pancreatic Cancer via Sponging miR-944

Supplementary Figure

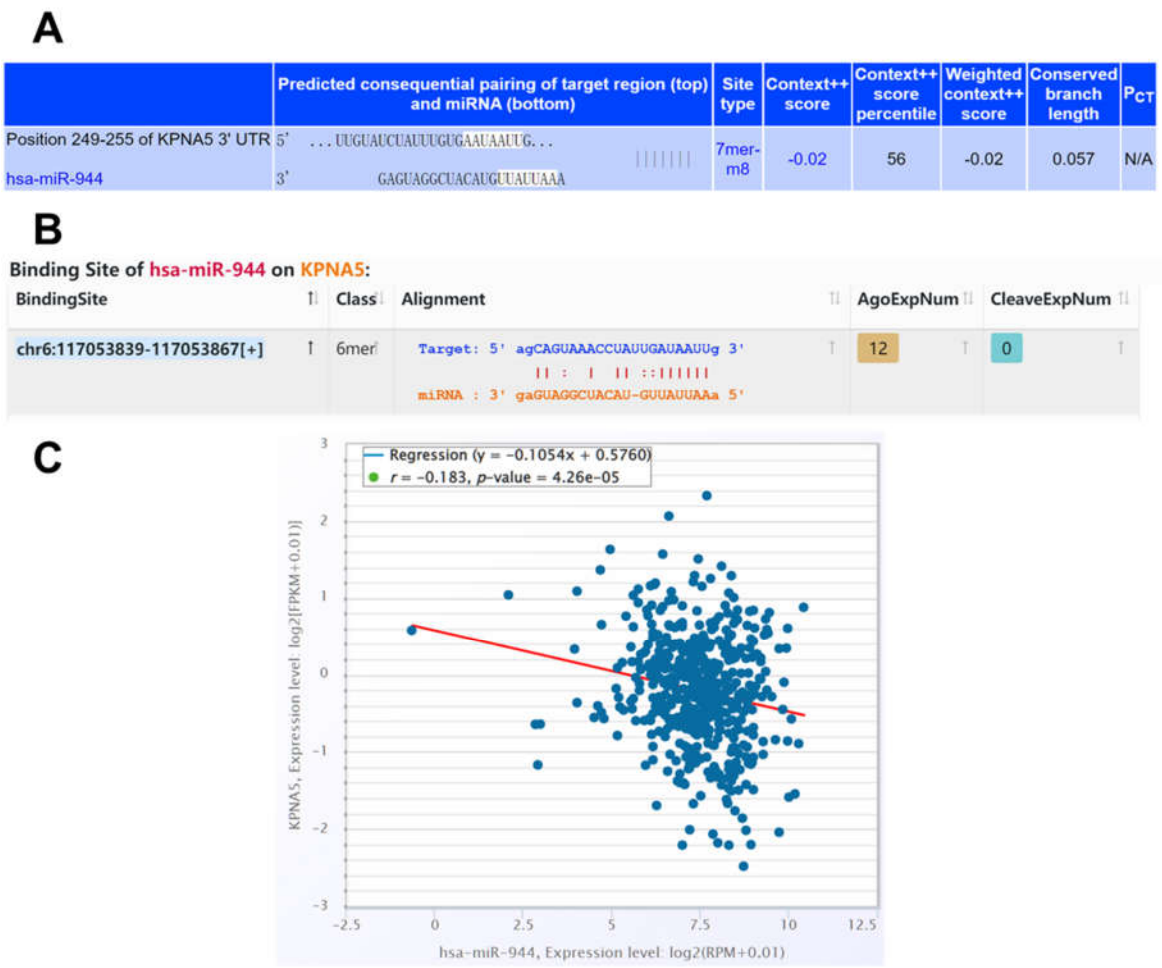

Figure S1. The interactive relationship between miR-944 and KPNA5. (**A**) Binding sites of miR-944 and lncRNA SNHG6, predicted by TargetScan. (**B**) Binding sites of miR-944 and lncRNA SNHG6, predicted by ENCORI, AgoExpNum, experimentally verified binding sites of KPNA5 to Ago proteins. (**C**) The correlation between expression of miR-944 and KPNA5, analyzed with ENCORI in pan-Cancer across 32 types of human cancers.
